# A new mechanism of respiratory syncytial virus entry inhibition by small-molecule to overcome K394R-associated resistance

**DOI:** 10.1128/mbio.01385-24

**Published:** 2024-08-20

**Authors:** Qiaoyun Song, Haoyue Zhu, Manlan Qiu, Jialiao Cai, Yun Hu, Haixia Yang, Shuwen Rao, Yaolan Li, Manmei Li, Lijun Hu, Shuqin Wang, Jian Hong, Wencai Ye, Heru Chen, Ying Wang, Wei Tang

**Affiliations:** 1 State Key Laboratory of Bioactive Molecules and Druggability Assessment, Jinan University, Guangzhou, China; 2 Guangdong Province Key Laboratory of Pharmacodynamic Constituents of TCM & New Drugs Research, College of Pharmacy, Jinan University, Guangzhou, China; 3 Center for Bioactive Natural Molecules and Innovative Drugs Research, College of Pharmacy, Jinan University, Guangzhou, China; 4 Department of Pathophysiology, School of Medicine, Jinan University, Guangzhou, China; Johns Hopkins Bloomberg School of Public Health, Baltimore, Maryland, USA; Georgia State University, Atlanta, Georgia, USA

**Keywords:** respiratory syncytial virus, antiviral, fusion protein, viral entry

## Abstract

Infection with respiratory syncytial virus (RSV) is a major cause of acute lower respiratory tract disease in young children and older people. Despite intensive efforts over the past few decades, no direct-acting small-molecule agents against RSV are available. Most small-molecule candidates targeting the RSV fusion (F) protein pose a considerable risk of inducing drug-resistant mutations. Here, we explored the *in vitro* and *in vivo* virological properties of the K394R variant, a cross-resistant mutant capable of evading multiple RSV fusion inhibitors. Our results demonstrated that the K394R variant is highly fusogenic *in vitro* and more pathogenic than the parental strain *in vivo*. The small molecule (2*E*,2′*E*)-*N*,*N*′-((1*R*,2*S*,3*S*)−3-hydroxycyclohexane-1,2-diyl)bis(3-(2-bromo-4-fluorophenyl) acrylamide) (CL-A3-7), a structurally optimized compound derived from a natural caffeoylquinic acid derivative, substantially reduced *in vitro* and *in vivo* infections of both wild-type RSV and the K394R variant. Mechanistically, CL-A3-7 significantly inhibited virus–cell fusion during RSV entry by blocking the interaction between the viral F protein and the cellular insulin-like growth factor 1 receptor (IGF1R). Collectively, these results indicate severe disease risks caused by the K394R variant and reveal a new anti-RSV mechanism to overcome K394R-associated resistance.

**IMPORTANCE:**

Respiratory syncytial virus (RSV) infection is a major public health concern, and many small-molecule candidates targeting the viral fusion (F) protein are associated with a considerable risk of inducing drug-resistant mutations. This study investigated virological features of the K394R variant, a mutant strain conferring resistance to multiple RSV fusion inhibitors. Our results demonstrated that the K394R variant is highly fusogenic in cell cultures and more pathogenic than the parental strain in mice. The small-molecule inhibitor CL-A3-7 substantially reduced *in vitro* and *in vivo* infections of both wild-type RSV and the K394R variant by blocking the interaction of viral F protein with its cellular receptor, showing a new mechanism of action for small-molecules to inhibit RSV infection and overcome K394R-associated resistance.

## INTRODUCTION

Respiratory syncytial virus (RSV) infection is a major cause of lower respiratory tract disease (LRTD) in young children and elderly individuals. In 2019, RSV was estimated to be responsible for more than 33 million episodes of acute LRTDs, 3.6 million hospitalizations, and 101,400 deaths among children under 5 years of age (1). RSV infects approximately 5%–10% of adults and causes an estimated 14,000 in-hospital deaths annually in those over 65 years of age (2, 3). Elderly patients with underlying heart and pulmonary diseases or compromised immune status are at a higher risk of RSV-related morbidity and mortality (4). Most RSV-related deaths are attributed to pediatric bronchiolitis and pneumonia (5). Over the past few decades, the development of anti-RSV drugs has never ceased because of the public health impact of this viral infection. To date, ribavirin remains the only small-molecule therapeutic drug available for RSV infection. However, ribavirin is a broad-spectrum antiviral drug that has not been recommended for treating RSV infections in the United Kingdom and the United States because of its associated adverse effects, such as potential teratogenicity and bone marrow suppression (6). Palivizumab (Synagis), a humanized monoclonal antibody (mAb) that targets the fusion (F) glycoprotein antigen of RSV, can be used to treat infants and children at high risk of RSV infection. Nevertheless, its application has been limited owing to its high cost and the requirement for repeated intramuscular injections over several months (7). Recently, another RSV F-specific mAb, nirsevimab (Beyfortus), was approved in the United States and Europe to prevent RSV-induced LRTD in infants during the first RSV season (8). Since the blockage of RSV entry with specific anti-F mAbs (e.g., palivizumab and nirsevimab) greatly reduces virus-related hospitalizations, targeting the RSV F protein with small-molecule inhibitors is expected to be an effective strategy for anti-RSV drug development.

RSV has three types of glycoproteins on the virion surface: the attachment glycoprotein (G), fusion glycoprotein (F), and the small hydrophobic (SH) protein. During the initial stage of RSV entry, the G protein adsorbs virions to the host cell surface (9). Thereafter, the viral F protein interacts with the host cell membrane and mediates the virus–cell fusion. This process involves the conformational rearrangement of RSV F from a metastable prefusion conformation to a highly stable postfusion state (10). Membrane merging enables the release of the viral nucleocapsid into the cytoplasm of the host cell. The expression of the viral F protein on the cell membrane triggers membrane fusion between neighboring cells, resulting in the formation of multinucleated syncytia. Some small-molecule inhibitors that target the viral F protein have been identified in recent studies, and several of these inhibitors, including rilematovir (JNJ-53718678), ziresovir (AK-0529), presatovir (GS-5806), and sisunatovir (RV-521), have shown therapeutic efficacy in individuals challenged with RSV (11–14). Up to now, none of them have been approved for the treatment of RSV infection. Moreover, escape mutations that confer resistance to these inhibitors occur frequently because of the rapid selection of resistant strains. Mutated RSV variants, particularly those carrying the K394R mutation in the viral F protein, exhibit substantial resistance to various fusion inhibitors (15–19).

We recently explored the mechanism underlying the cross-resistance of the K394R variant to RSV fusion inhibitors and demonstrated that the K394R mutation in the F protein enhanced viral fusogenicity (18). Previous studies have revealed that the fusogenicity of some enveloped viruses is closely correlated with their pathogenicity and ability to evade immune surveillance *in vivo*. For example, the RSV F mutant A2-line 19F with high fusion activity induces severe lung histopathology, weight loss, and high viral loads in mice (20). The P681R mutation in the spike (S) protein of the SARS-CoV-2 Delta variant increases viral fusogenicity in cell cultures, enhances viral pathogenicity in the lungs of hamsters, and confers resistance to neutralizing antibodies elicited by vaccination (21). In contrast, the Omicron sublineages of SARS-CoV-2 exhibit lower fusogenicity and are less pathogenic than the ancestral viruses (22). Positive correlations between fusogenicity and severity of viral pathogenicity have also been reported in infections with other enveloped viruses such as measles and HIV-1 (23–25). Generally, higher viral fusogenicity is associated with an increased capacity for cell-to-cell transmission, which is considered an efficient strategy for virions to evade host immunosurveillance *in vivo*, particularly extracellular neutralization by antibodies. The K394R mutation in the RSV F protein confers cross-resistance to fusion inhibitors, including several inhibitor candidates currently undergoing clinical trials. The virological characteristics of the K394R variant, such as its *in vivo* pathogenicity and resistance to neutralizing antibodies elicited by vaccination, remain unclear.

In this study, we demonstrated that the K394R variant with higher fusogenicity is more pathogenic than the parental virus in the respiratory tract and lungs of mice and showed cross-resistance to viral fusion inhibitors as well as to prefusion RSV F antigen-induced neutralization. These observations suggest that potential risks to public health, including antiviral resistance and more severe manifestations, may occur when K394R-related fusion inhibitors are used in the clinical treatment of RSV infections. To address this concern, a small-molecule inhibitor CL-A3-7, chemically named (2*E*,2′*E*)-*N*,*N*′-((1*R*,2*S*,3*S*)−3-hydroxycyclohexane-1,2-diyl)bis(3-(2-bromo-4-fluorophenyl)acrylamide), capable of inhibiting infections of the K394R variant and wild-type RSV was identified via structure-optimization from 3,4-DCQAME, a natural fusion inhibitor of RSV identified in our previous studies (26, 27). Furthermore, the underlying mechanism of action and *in vivo* anti-RSV activity of CL-A3-7 were also investigated in this study.

## RESULTS

### The K394R mutation enhances RSV fusogenicity *in vitro*

**Fig 1:**
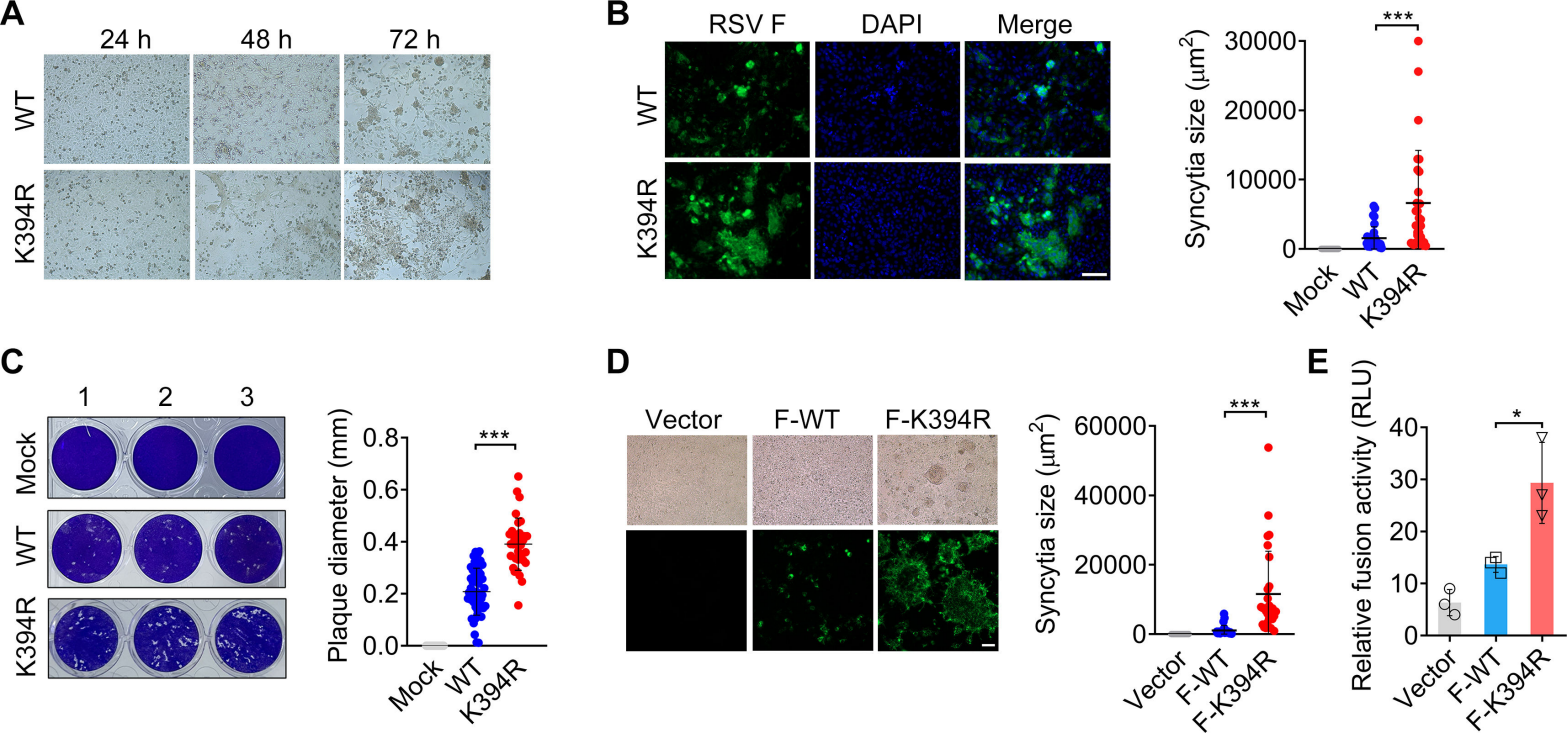
Virological characteristics of the K394R variant *in vitro*. (A) Representative images of syncytia in the WT- and K394R-infected HEp-2 cells. (B) Left panel, representative immunofluorescence staining of syncytia in the WT- and K394R-infected HEp-2 cells. Bar, 100 µm. Right panel, the area of syncytia in the infected cells was measured using ImageJ software. (C) Plaques formed in Mock-, WT-, or K394R-infected cells as determined by the plaque assay. Left panel, representative images of plaques. Right panel, the dots in data indicate the plaque diameter. (D) Left panel, representative images of syncytia formation in HEK293T cells transfected with WT F or F-K394R mutation. Bar, 50 µm. Right panel, syncytial area in transfected cells is indicated. (E) Membrane fusion activities in the transfected cells were determined by a luciferase-based assay. (B–E) Data are mean ± standard deviation (SD). The two-tailed Student’s *t* test was used to measure the statistical difference between compared groups. *, *P* < 0.05; ***, *P* < 0.001.

To investigate the effect of the K394R mutation on the virological features of RSV, we tested the ability of wild-type (WT) RSV and the K394R variant (K394R) to infect four human epithelial cell lines that are susceptible and permissive to RSV. The cell lines A549, BEAS-2B, and 16HBE are derived from the human respiratory system and are the main targets of RSV infection. As shown in Fig. S1, both WT and K394R strains are efficiently grown in these cells. In HEp-2 cells, the K394R variant showed a slightly slower growth rate than the WT. At 72 h post-infection (hpi), the viral RNA level in the K394R variant-infected HEp-2 cells was 2.2-fold lower than that in WT-infected cells. In contrast, the growth kinetics of the K394R and WT were comparable in A549, BEAS-2B, and 16HBE cells (Fig. S1B through D). Although the viral RNA level and syncytia numbers in the K394R variant-infected cells were lower or comparable to those in cells infected with the WT (Fig. 1A), multinucleated and larger syncytia were formed in cells infected with the K394R variant (Fig. 1A through C). The average diameter of the plaques generated in the K394R variant-infected cells was 1.9-fold larger than that in cells infected with the WT (Fig. 1C). Moreover, larger syncytia or multinuclear fused cells were observed in the cells transfected with plasmids encoding RSV F carrying the K394R mutation (Fig. 1D). While transfection with plasmids expressing WT F caused the formation of some fused cells, large syncytia between adjacent cells were not observed (Fig. 1D). Subsequently, cell–cell fusion activity in these transfected cells was measured using a luciferase-based fusion assay (18). F-K394R-transfected cells exhibited greater fusion activity than the cells transfected with WT F (Fig. 1E). These results suggest that the K394R mutation in the RSV F protein enhances viral fusogenicity and leads to greater syncytial formation than that observed in infection with the WT.

### Virological features of the K394R variant *in vivo*

**Fig 2:**
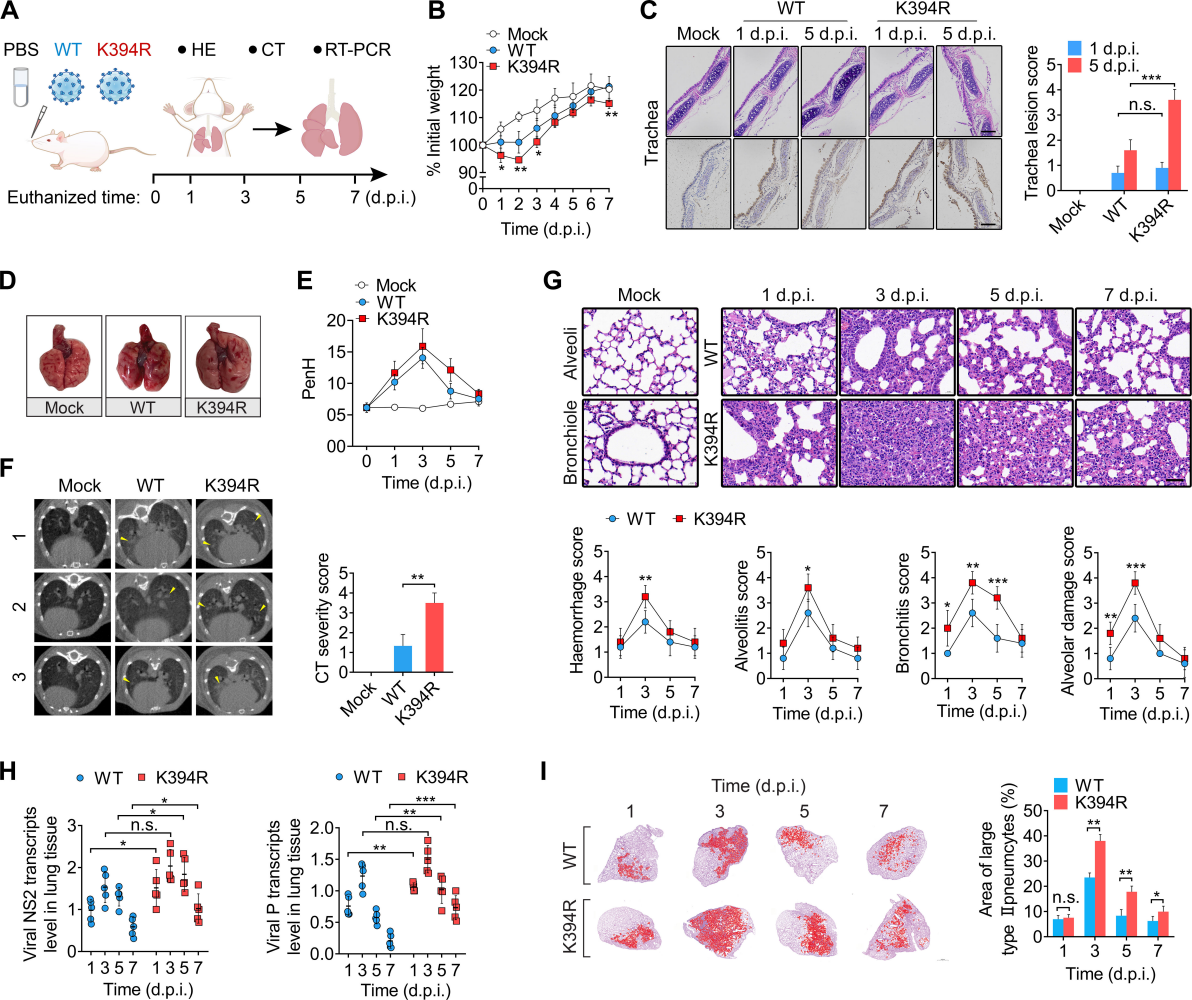
*In vivo* pathogenicity of the K394R variant. (A) Schematic showing experimental design. (B) Body weight changes in mock-, WT-, and K394R variant-infected mice. Data are mean ± SD (*n* = 6). Statistically significant differences in the comparison between the K394R variant- and WT-infected mice at each time point are indicated by asterisks. (C) Left panel, representative H&E staining (top) and immunohistochemical staining (bottom) images of trachea sections of mice. Right panel, lesion severity score of respiratory tracheas of mice. Bars, 100 µm. Data are mean ± SD (*n* = 5). (D) Representative images of lung organs of mice at 5 dpi are shown. (E) Enhanced pause (PenH) of the mice is indicated. Data are mean ± SD (*n* = 6). (F) Left panel, micro-CT scans of the lungs of mice (*n* = 3) at 5 dpi. Lung abnormalities of infected mice are indicated as yellow asterisks. Right panel, analysis of the CT lung severity score. Data are mean ± SD (*n* = 3). (G) Top panel, representative H&E staining images of lung tissues of mice. Bottom panel, histopathological score of lung lesions. Representative features of lung lesions were analyzed. Data are mean ± SD (*n* = 5). Statistically significant differences in comparison between WT- and K394R variant-infected mice are indicated. Bar, 50 µm. (H) Viral RNA loads in the lung tissues of infected mice are shown. The transcript levels of viral non-structural protein 2 (NS2) and phosphoprotein (P) in the lung homogenates of WT- or K394R-infected mice were determined by RT-PCR assay. Data are mean ± SD (*n* = 4 or 5). (I) Left panel, the inflammatory infiltrations with type II pneumocytes in the representative lung lobes are indicated in red color. Right panel, the percentage of the area covered by type II pneumocytes to the total area in the lung sections was calculated. Data are mean ± SD (*n* = 5). (B, C, F–I). An unpaired two-tailed *t* test was used for two groups comparisons. *, *P* < 0.05; **, *P* < 0.01; ***, *P* < 0.001; n.s., no significant difference.

The K394R mutation is known to enhance RSV fusogenicity in cell culture. However, the effects of this mutation on viral pathogenicity remain unclear. To investigate the *in vivo* pathogenic characteristics of the K394R variant, BALB/c mice were intranasally inoculated with the WT or the K394R variant (Fig. 2A). Compared with mock-infected mice, both WT- and K394R-infected mice showed slower weight gain beginning 1 day after infection (Fig. 2B). K394R-infected mice showed greater weight loss than WT-infected mice over the duration of the infection. From 1 to 3 days post-infection (dpi), the average weight of K394R variant-infected mice was significantly lower than that of WT-infected mice, and the weight discrepancy between the two groups peaked at 2 dpi (Fig. 2B). Although the average weight of mice from all groups increased from 3 to 6 dpi, the final average weight of the K394R variant-infected mice at 7 dpi was significantly lower than those of the mock- and WT-infected mice (Fig. 2B).

Hematoxylin and eosin (H&E) staining revealed necrosis and degeneration of mucosal epithelial cells in the tracheas of infected mice at 1 or 5 dpi (Fig. 2C, top left). The histopathological changes in the tracheal tissues of K394R-infected mice were more severe than those in WT-infected mice (Fig. 2C, right panel). To detect virion production, immunohistochemical (IHC) staining for viral antigens was performed using tracheas from the respiratory tract of mice. On days 1 and 5 after viral infection, substantial numbers of virions were observed in the epithelial cells around the mucosal surface of the trachea (Fig. 2C, bottom left panel). Viral antigen levels of WT-infected mice were comparable to those of K394R-infected mice at 1 dpi and decreased at 5 dpi. In contrast, viral antigen production in the tracheal epithelial cells of K394R-infected mice remained relatively high at 5 dpi (Fig. 2C, bottom left panel).

Moreover, the morphological characteristics of the lungs showed that both WT and K394R variant infections led to apparent pathological changes (Fig. 2D). As compared with WT-infected mice, mice infected with the K394R variant exhibited extensive lesions and more severe pulmonary edema (Fig. 2D). Next, we evaluated the pulmonary function of mice from each group. The enhanced pause (PenH), a surrogate marker of airway obstruction or bronchoconstriction, was measured using whole-body plethysmography. Both WT and K394R variant infections increased PenH levels in lungs (Fig. 2E). The PenH values of the K394R variant-infected mice were higher than those of the WT-infected mice at all time points (Fig. 2E). Micro-computed tomography (CT) analysis further demonstrated lung abnormalities in all infected mice at 5 dpi (Fig. S2). The abnormalities, such as pulmonary consolidation with inflammatory infiltrates, occurred in the lung tissues of both WT- and K394R variant-infected mice (Fig. 2F, left). In comparison with mice infected with the WT, those infected with the K394R variant showed a higher CT severity score (Fig. 2F, right panel).

In addition, the H&E stained images demonstrated the pathological changes in the lung tissues of mice (Fig. 2G). In mock-infected mice, pulmonary alveoli, capillary endothelial cells, bronchi, and bronchioles were maintained in a normal state, and no inflammatory cell infiltration was observed in the lung tissues of these mice (Fig. 2G). At 1 dpi, mild bronchitis and alveolitis were observed in the lungs of WT- and K394R-infected mice. Alveolar damage and severe inflammatory cell infiltration in the bronchi and alveoli in the lungs were observed at 3 dpi with the K394R variant. (Fig. 2G, top panel). In contrast, the WT-infected mice showed fewer pathological changes including bronchitis and alveolitis. Acute inflammatory responses in WT-infected mice, such as bronchitis, alveolitis, and hemorrhage, were sharply attenuated after 5 dpi, whereas the inflammatory responses and histopathological changes remained at a relatively high level at 5 dpi in the lung tissues of mice infected with the K394R variant (Fig. 2G, bottom panel).

Next, we assessed virus production in the trachea and lungs of infected mice. As shown in Fig. 2H and Fig. S3, the dynamics of viral RNA loads in the tracheas and lung tissues of K394R-infected mice were different from those in WT-infected mice. Viral RNA levels in WT-infected mice peaked at 3 dpi and sharply decreased after 5 dpi. In contrast, at 5 and 7 dpi, the viral RNA loads in the tracheas and lungs of K394R variant-infected mice were more stable and higher than those of WT-infected mice (Fig. 2H; Fig. S3). Subsequently, the hyperplasia area of type II large pneumocytes was quantitatively analyzed. At 3 dpi, all infected mice showed an increase in hyperplasia of large type II pneumocytes, which sharply decreased from 5 to 7 dpi (Fig. 2I). Throughout infection period, the areas covered by large type II pneumocytes in the lung tissues of K394R-infected mice were significantly larger than that in WT-infected mice (Fig. 2I). Taken together, these results suggest that the K394R variant is more pathogenic and causes more prolonged infection and severe pathology than WT in mice.

### Cross-resistance conferred by the K394R mutation

To assess the resistance of the K394R variant to RSV fusion inhibitors and neutralizing antibodies induced by the viral prefusion F antigen, the growth kinetics of the WT and the K394R variant were determined. In HEp-2 cells treated with RSV fusion inhibitors or an RSV nucleoprotein (N) inhibitor (Zelicapavir), the virus yield of WT virions was suppressed to a relatively low level (Fig. S4A). In contrast, the K394R variant was resistant to these fusion inhibitors but remained sensitive to the N protein inhibitor (Fig. S4B). All the tested inhibitors exhibited potent inhibitory effects on WT infection at nanomolar concentrations, whereas they did not block the infection and growth of the K394R variant, even at concentrations 20-fold higher than the half-maximal inhibitory concentration (IC_50_; Fig. S4B). In particular, virus yields of the K394R variant in the BMS-433771- and JNJ-53718678-treated cells were comparable to those in untreated cells. These results are consistent with previous reports indicating that the K394R mutation in RSV F protein confers the exceptional resistance to the fusion inhibitors tested in this study.

To assess the effect K394R mutation on vaccine-induced antibody neutralization, the sera of BALB/c mice were collected after immunization with DS-Cav1, a prefusion F-stabilized candidate vaccine, to neutralize WT and K394R-mutated viruses in HEp-2 cells. A neutralization assay using the mouse sera after two or three rounds of DS-Cav1 vaccination demonstrated that the K394R variant was less sensitive to the neutralizing antibodies induced by DS-Cav1 (Fig. S4C and D). As a control, WT infection of HEp-2 cells was potently inhibited by diluted sera from the vaccinated mice in a concentration-dependent manner.

### CL-A3-7 inhibits infections of the WT and the K394R variant

**Fig 3:**
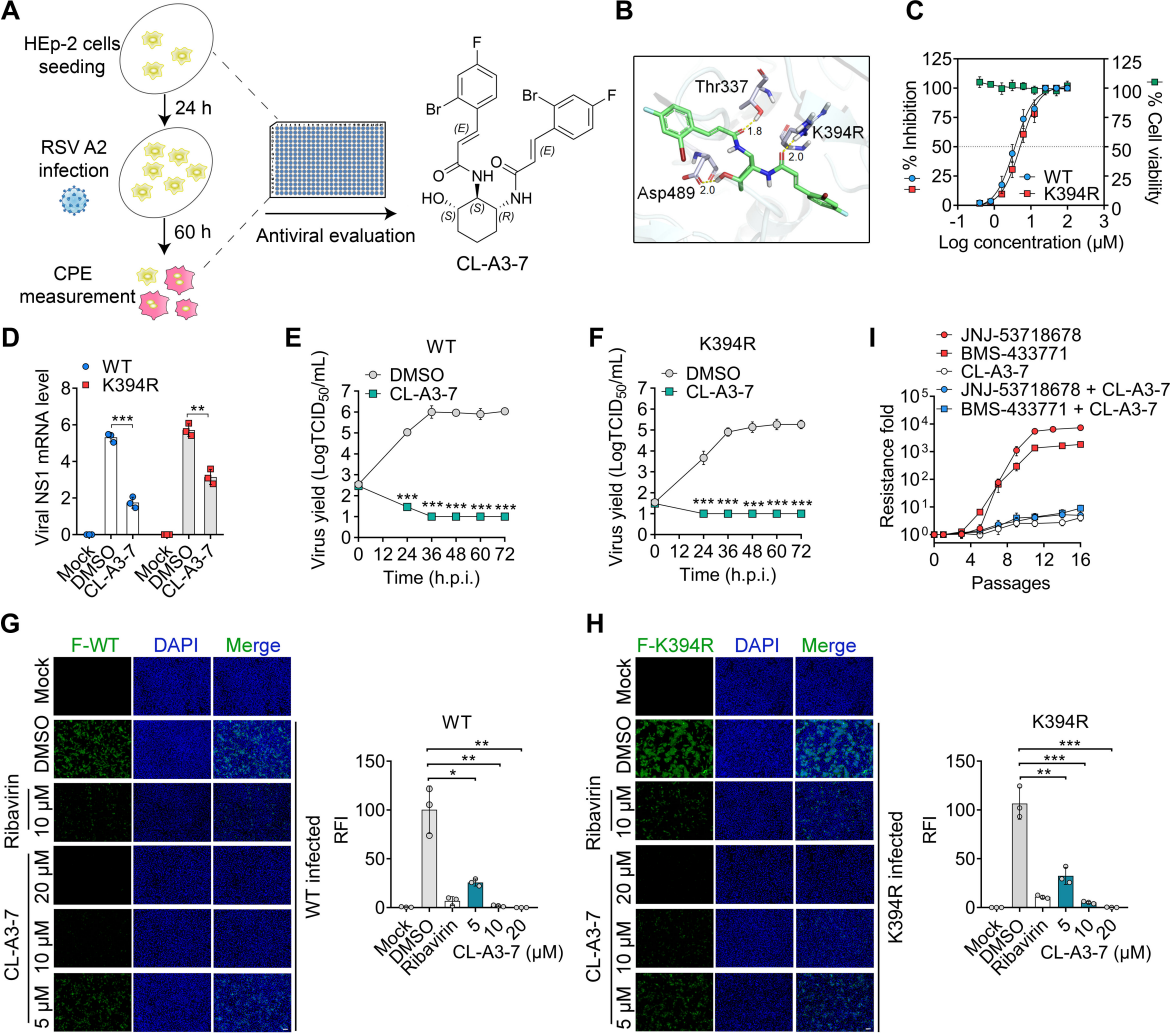
*In vitro* anti-RSV activity of CL-A3-7. (A) A schematic of the analysis of anti-RSV activity. (B) 3D structural view of CL-A3-7 bound to residues of prefusion RSV F (PDB 7UJA) with the K394R mutation. (C) Effects of CL-A3-7 on cell viability and viral infection in HEp-2 cells were determined by CCK-8 and cytopathic effect (CPE) inhibition assays. Values are normalized to those obtained for DMSO-treated cells and presented as mean ± SD, *n* = 3 biological replicates. (D) Viral NS1 mRNA level in HEp-2 cells in the presence of CL-A3-7 (10 µM) or DMSO. Growth kinetics of WT (E) and K394R variant (F) in HEp-2 cells treated with CL-A3-7 (40 µM) or DMSO. Data are mean ± SD, *n* = 3 biological replicates. Statistically significant differences in the comparison between DMSO-treated and WT-treated cells at each time point are indicated by asterisks. (G, H) Representative images of immunostaining of the viral F protein in WT- and K394R-infected cells at 48 hpi. Relative immunofluorescent intensity (RFI) in each group of cells was analyzed using ImageJ software (G and H, right). Bar, 100 µm. (I) HEp-2 cells were inoculated with RSV A2 in the presence of increasing concentrations of the inhibitors. The starting concentrations of CL-A3-7, BMS-433771, and JNJ-53718678 were 2 µM, 20 nM, and 2 nM, respectively. Supernatants from cell cultures exhibiting cytopathic effects were harvested for the next passage of infection. For the combination treatments, CL-A3-7 was used at 8 µM after the third passage. Resistance fold was calculated based on the IC_50_ changes. (D–H) Data are mean ± SD, *n* = 3 biological replicates. The two-tailed Student’s *t* test was used to measure the statistical difference between groups. *, *P* < 0.05; **, *P* < 0.01; ***, *P* < 0.001.

Infection with the K394R variant resulted in more severe pathogenicity in the lungs of mice than WT infection. Moreover, the K394R-mutated virus exhibited substantial resistance to various therapeutic inhibitor candidates. These findings highlight the importance of identifying alternative inhibitors to combat K394R mutants. Previously, we identified 3,4-DCQAME as an RSV entry inhibitor that blocked viral fusion with the cell membrane (26, 27). Similar to the RSV fusion inhibitors described above, 3,4-DCQAME elicited a K394R mutation in the viral F protein. To optimize the binding mode of the molecule with the trimeric prefusion RSV F and confer the ability to inhibit the viral variant, the compound CL-A3-7, which is predicted to directly interact with three residues (Thr337 [T337], Arg394 [R394], and Asp489 [D489]) in the K394R-mutated prefusion F protein, was designed (Fig. 3A and B), and subsequently synthesized by structural modification of 3,4-DCQAME (Fig. S5A). Based on the principles of molecular simplification and bioelectronic isoarrangement (Fig. S5A), the hydroxyl and methoxycarbonyl groups at the C1 position of 3,4-DCQAME were removed and simplified. Meanwhile, the 3,4-dicaffeoyl-oxyl groups were bioelectro-isoarranged to 3,4-bis(3-(2-bromo-4-fluorophenyl) acrylamido) amino groups.

Four sets of experiments were conducted to assess the inhibitory effects of CL-A3-7 on WT and K394R variant infections. First, viral infections in HEp-2, 16HBE, and BEAS-2B cells were determined. CL-A3-7 potently inhibited infections of both the WT virus and the K394R variant (Fig. S5B through D) and reduced viral infection in a concentration-dependent manner without obvious cytotoxicity (Fig. 3C). Next, the level of viral NS1 mRNA in RSV-infected cells was determined using reverse transcription-quantitative polymerase reaction (RT-qPCR). Treatment with CL-A3-7 (10 µM) significantly reduced the viral RNA loads in both WT-infected and K394R-infected cells (Fig. 3D). Third, the viral growth kinetics were determined at indicated interval times post-inoculation. In CL-A3-7-treated cells, reductions of more than 107,000-fold and 14,000-fold in viral titers of the WT and the K394R variants, respectively, were observed at 72 hpi (Fig. 3E and F). In addition, the immunofluorescence assays further indicated that viral protein levels in both WT- and K394R-infected cells significantly decreased after treatment with CL-A3-7 in a concentration-dependent manner (Fig. 3G and H). As a control, ribavirin exhibited a weaker inhibitory effect on viral protein expression. Importantly, no resistant RSV were generated after culturing the virus with continuous CL-A3-7 treatment (Fig. 3I). Consistent with the results from previous reports, the RSV fusion inhibitors JNJ-53718678 and BMS-433771 induced drug resistance after incubation with RSV for 6–12 passages (Fig. 3I). Notably, combined treatment of these fusion inhibitors with CL-A3-7 dramatically reduced the appearances of antiviral resistance (Fig. 3I).

### Anti-RSV mechanism of CL-A3-7

We conducted a time-of-addition assay to determine which step in the RSV lifecycle is inhibited by CL-A3-7. In this assay, HEp-2, 16HBE, and BEAS-2B cells were inoculated with the WT or the K394R variant and treated with CL-A3-7 at the indicated time points before, during, or after viral entry into cells. Substantial reductions of viral infections were observed in the cells treated with CL-A3-7 before or during viral entry (Fig. S5C), whereas the virus yields were rapidly increased when the CL-A3-7 were added to cells later than 2 hpi (Fig. S5D). These results were consistent with the observation that the anti-RSV efficacy of CL-A3-7 was sharply attenuated after viral entry (Fig. S5D), suggesting that CL-A3-7 blocked the entry of RSV into cells.

**Fig 4:**
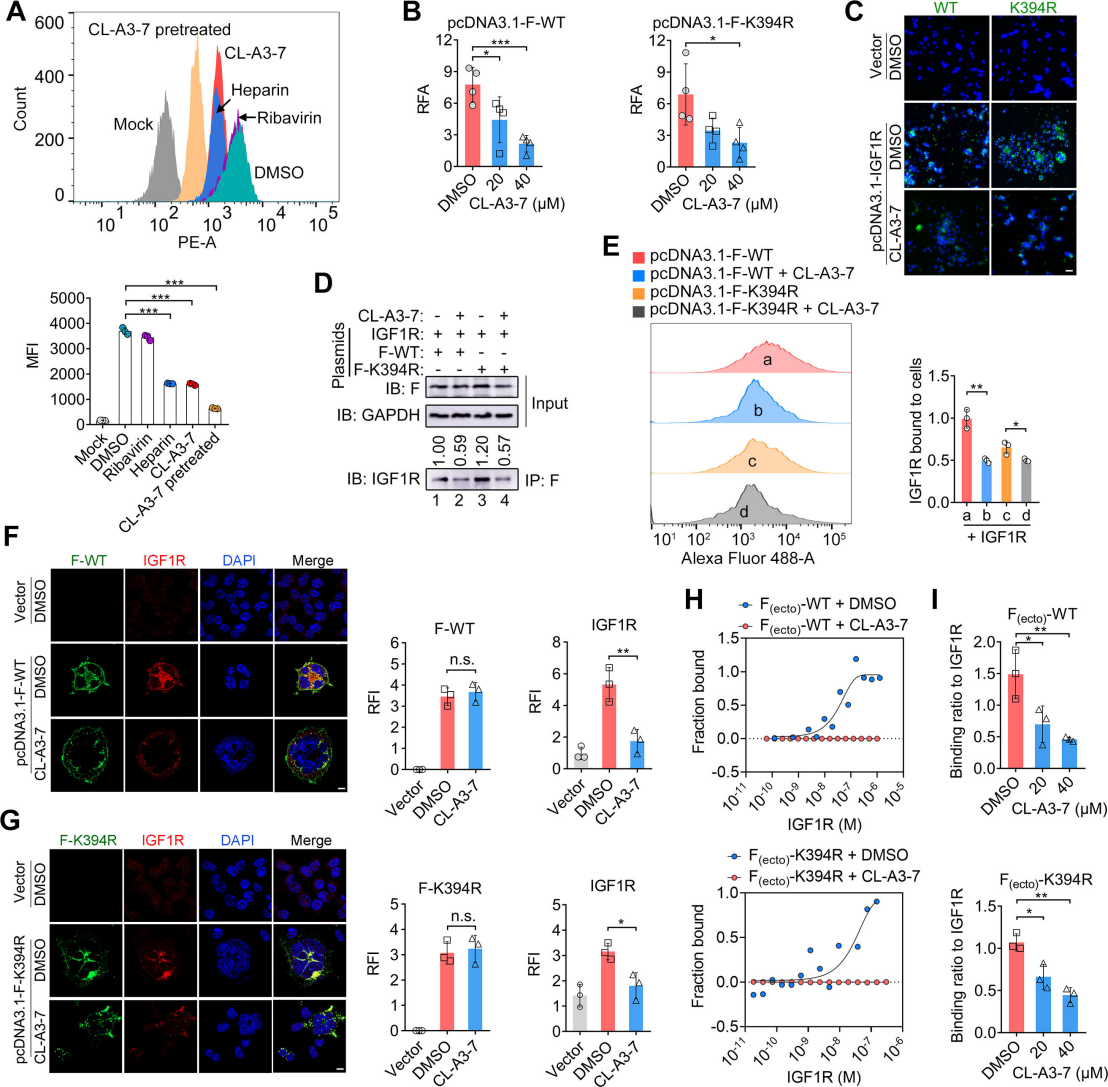
Action mechanism of CL-A3-7 against RSV. (A) HEp-2 cells were inoculated with octadecyl rhodamine B (R18)-labeled virions in the presence of CL-A3-7 (40 µM), ribavirin (40 µM), or heparin (4 µM) for 2 h at 37°C. Virus-cell fusion was measured by flow cytometry (FCM). Representative histograms of the FCM data (top panel) and the mean fluorescence intensity (MFI; bottom panel) are indicated. (B) HEK293T cells were transfected with F-WT or F-K394R plasmids and then treated with CL-A3-7 (20 or 40 µM) or DMSO. At 36 h after transfection, the cell–cell fusion activity was determined using a dual-luciferase system. RFA, relative fusion activity. (C) C636 mosquito cells were transfected with plasmids expressing IGF1R and treated with CL-A3-7 or DMSO, and then inoculated with the WT or K394R variant. Representative images of cells with DAPI (blue) and anti-RSV F (green) staining are shown. Scale bar, 20 µm. (D) Western blot and immunoprecipitation analysis of the binding of IGF1R with F-WT or F-K394R. The IGF1R band intensity after immunoblotting was calculated. (E) Binding of IGF1Rs on the cell surface in F-WT- or F-K394R- transfected cells. CL-A3-7 (20 µM). Representative FCM histograms (left panel) and the MFI correlated with the IGF1Rs on the cell surface were analyzed using FlowJo v.10.0 (right panel). (F, G) Left panel, the representative images of colocalization between IGF1R and F-WT (top) or between IGF1R and F-K394R (bottom). Right panel, viral F- and IGF1R-associated relative fluorescence intensity (RFI) are indicated. HEK293T cells were transfected with F-WT or F-K394R plasmids and then treated with IGF1R (2 µg) in the presence of CL-A3-7 (40 µM) or DMSO, followed by incubation with anti-IGF1R and anti-RSV F antibodies, and then stained with Alexa Fluor 488 and 594-conjugated antibodies, respectively, and subsequently detected with laser scanning microscope. Scale bar, 10 µm. (H) The interactions of IGF1R with F_(ecto)_- or with F_(ecto)_-K394R were determined by MST assay. The RED-NHS dye-labeled F_(ecto)_- and F_(ecto)_-K394R were premixed with CL-A3-7 (40 µM) and then treated with IGF1R protein. (I) Binding of IGF1R with F_(ecto)_- or F_(ecto)_-K394R in the presence of CL-A3-7 or DMSO was determined by ELISA. (A, B, E–G, I) Data are mean ± SD, *n* = 3 or 4 biological replicates. The two-tailed Student’s *t* test was used to measure the statistical difference between groups. *, *P* < 0.05; **, *P* < 0.01; ***, *P* < 0.001; n.s., no significant difference.

Next, we performed viral attachment and membrane fusion assays to determine the step of viral entry that was inhibited by CL-A3-7. The number of virions adsorbed on the cell surface was not disrupted by treatment of CL-A3-7 even at a concentration 10-fold greater than the IC_50_ (Fig. S5E and F). In contrast, the positive control heparin potently inhibited the attachment of virions to the cell surface. In the viral fusion assay, purified virions were labeled with octadecyl rhodamine B chloride (R18), a lipophilic dye that is self-quenched at a relatively high concentration on the surface of virions and eliminates this effect during viral fusion (18, 27). We observed that diffusion of the R18 probes from the virion surface to the cell membrane was blocked by CL-A3-7 (Fig. 4A). As a negative control, ribavirin did not block the diffusion of R18 probes, as expected (Fig. 4A). Furthermore, the luciferase-based fusion assay demonstrated that cell–cell fusion activity in F-WT-transfected or F-K394R-transfected cells was suppressed by CL-A3-7 (Fig. 4B). These results suggest that CL-A3-7 inhibits virus–cell and cell–cell fusion mediated by the RSV F protein.

Insulin-like growth factor 1 receptor (IGF1R) was recently identified as an RSV entry receptor that interacts with viral F protein during RSV entry (28). In our experiments, RSV infection of human epithelial cells was inhibited by the soluble IGF1R protein, as evidenced by fewer syncytia and lower expression levels of viral proteins in IGF1R-treated cells than untreated cells (Fig. S6B and C). We previously demonstrated that 3,4-DCQAME, the parent compound of CL-A3-7, blocked the interaction of RSV F protein with the target cell membrane (27). Moreover, virus–cell and cell–cell fusion mediated by the RSV F protein can be disrupted by CL-A3-7. To test whether CL-A3-7 blocked the association of the F protein with the cellular receptor IGF1R, the C636 mosquito cell line that is non-permissive to RSV infection was transfected with plasmids expressing IGF1R. As shown in Fig. 4C, overexpression of human IGF1R conferred RSV the ability to infect C636 cells, consistent with previous reports that IGF1R serves as a receptor during RSV entry. Both WT and K394R infections in IGF1R-transfected cells were suppressed by CL-A3-7 (Fig. 4C). Co-immunoprecipitation assays further suggested that the interactions between IGF1R and F-WT or F-K394R were inhibited by CL-A3-7 (Fig. 4D). A significant reduction of purified IGF1R on the cell surface was observed in F-WT- and F-K394R-transfected cells treated with CL-A3-7 (Fig. 4E). Furthermore, co-localization of IGF1R and F-WT or F-K394R was blocked by CL-A3-7 (Fig. 4F and G). Using microscale thermophoresis (MST), we detected a relatively high binding affinity between IGF1R and the ectodomains of F-WT or F-K394R, with dissociation constant (*K*_*D*_) values of 10.97 and 12 nM, respectively (Fig. 4H). The interaction of IGF1R with F_(ecto)_-WT or F_(ecto)_-K394R was interrupted by CL-A3-7, consistent with the results of the enzyme-linked immunosorbent assay (ELISA) (Fig. 4I). Taken together, these results suggest that CL-A3-7 inhibits RSV entry by blocking the interaction of viral F protein with the cellular IGF1R.

Previous reports described that RSV variants with a D489Y mutation in the viral F protein showed cross-resistance to several fusion inhibitor candidates, including JNJ-53718678, RV-521, and AK-0529 (18, 29, 30). We subsequently tested whether the cell-cell fusion mediated by F-D489Y could be inhibited by CL-A3-7. A large number of syncytia was observed in DMSO-treated cells, as well as in cells that were treated with JNJ-53718678, RV-521, or AK-0529 at concentrations 20-fold higher than their IC_50_ values. In contrast, hardly any syncytia were observed in CL-A3-7-treated cells (Fig. S7). These results suggest that distinct antiviral mechanism of CL-A3-7 confer its ability to resist other RSV fusion inhibitors' resistant mutation, including K394R and D489Y.

### *In vivo* anti-RSV efficacy of CL-A3-7

**Fig 5:**
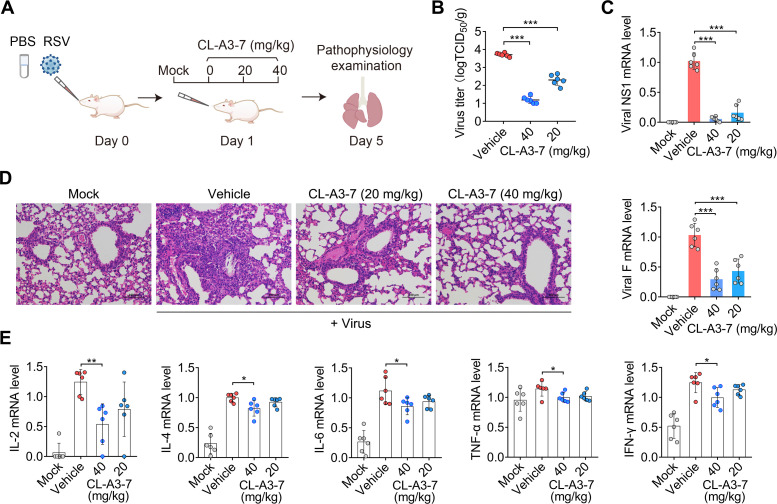
*In vivo* efficacy of CL-A3-7 against the K394R mutant. (A) Schematic design for mice study. (B) Viral loads in the lung homogenates of mice treated with vehicle or CL-A3-7 (40 and 20 mg/kg) are shown. (C) Detection of viral mRNAs (F and NS1) levels in the lung homogenates by quantitative RT-qPCR assay. (D) Representative images of H&E staining of the lung tissue sections at 5 dpi. Bars, 100  µm. (E) The mRNA levels of inflammatory cytokines (IL-2, IL-4, IL-6, TNF-α, and IFN-γ) in the lung tissues of mice are indicated. (B–E) Data are mean ± SD (*n* = 6). The two-tailed Student’s *t* test was used for two groups comparison test, *, *P* < 0.05; **, *P* < 0.01; ***, *P* < 0.001.

To evaluate the *in vivo* anti-RSV activity of CL-A3-7, BALB/c mice were inoculated intranasally with the K394R variant and treated once daily with CL-A3-7, beginning at 24 h post-inoculation. Lung tissues from each group of mice were collected at 5 dpi (Fig. 5A). At 5 dpi, 330- and 24-fold reductions in viral titers were observed in the lung tissues of mice treated with 40 and 20 mg/kg CL-A3-7, respectively (Fig. 5B). Moreover, the mRNA levels of the viral NS1 and F genes in the lung tissues of CL-A3-7-treated mice were significantly lower than those in vehicle-treated mice (Fig. 5C). Upon treatment with CL-A3-7 (40 mg/kg), a 19-fold reduction in the mRNA level of the viral NS1 gene was observed in the lung tissues of the infected mice at 5 dpi (Fig. 5C).

Inflammatory infiltrations, including bronchitis and alveolitis, alveolar damage, and crushed nuclear debris around the bronchioles, were observed in the lung tissues of infected mice (Fig. 5D). Treatment with CL-A3-7 (40 and 20 mg/kg) substantially reduced acute inflammatory responses, including bronchitis and alveolitis, in the lung tissues of mice and resulted in fewer pathological changes than those observed in vehicle-treated mice (Fig. 5D). The immune response caused by RSV infection enhanced the expression and secretion of inflammatory cytokines in lung tissues, such as interleukin-2 (IL-2), IL-4, IL-6, tumor necrosis factor alpha (TNF-α), and gamma interferon (IFN-γ). The levels of all the tested inflammatory-related cytokines in the lung tissues of mice were reduced by treatment with CL-A3-7 (40 and 20 mg/kg) (Fig. 5E). Taken together, these results suggest that CL-A3-7 blocks RSV infection *in vivo*.

## DISCUSSION

The RSV F glycoprotein is the main target of *in vivo* neutralizing antibodies and could serve as a major antigen for RSV vaccine construction. Thus far, most RSV vaccines and vaccine candidates, such as Abrysvo, Arexvy, and DS-Cav1, have been designed on the basis of the prefusion-stabilized conformation of the F protein. The RSV F protein has also been deemed an attractive target for the development of anti-RSV therapeutics. In recent years, various humanized monoclonal antibodies (hmAbs) and small-molecule compounds capable of blocking virus–cell and cell–cell fusion mediated by the RSV F protein have been identified. Several of them (e.g., BMS-433771, JNJ-53718678, AK-0529, RV-521, and GS-5806) exhibit potent anti-RSV activity in animal models or patients challenged with RSV. However, these virus-targeted inhibitors of RSV, including neutralizing antibodies and small molecules, are associated with a high risk of selecting resistant mutants because of the high mutation rates of the viral genome under selection pressures. In particular, the K394R mutation in the RSV F protein confers broad-spectrum resistance to various fusion inhibitors. It is therefore important to investigate the potential antiviral resistance and severe disease risk associated with the K394R variant, and to identify inhibitors capable of blocking the *in vivo* infection caused by this variant.

The correlation between fusogenicity in cell culture and pathogenicity *in vivo* can vary among enveloped RNA viruses. For instance, measles viral variants containing several mutations in fusion protein that exhibit high fusogenicity cause fatal subacute sclerosing panencephalitis (subsp.) or promote the virions spread in human neuronal cells (23, 24). As compared with wild-type viruses, measles variants with higher fusogenicity display diminished efficiency in cell cultures but favor the viral propagation in human brains (31). An HIV mutant with higher fusogenicity than the parental virus did not promote viral replication in cell culture (32). Nevertheless, the higher fusogenicity of mutated HIV viruses is associated with more severe illness in patients, including HIV-induced encephalitis (33). Our observations in the present study are similar to the findings described in these reports. The K394R variant with higher fusogenicity did not increase the efficiency of viral growth *in vitro* but enhanced virus-induced pathological changes in the trachea and lung tissues of mice. The K394R is a cross-resistance mutation for RSV fusion inhibitors with diverse chemical structures. Enhanced pathogenicity caused by the K394R mutation may lead to antiviral resistance and more severe disease manifestations when these fusion inhibitors are employed in clinical therapy for RSV infection.

Higher viral fusogenicity is generally correlated with the increased viral ability for cell–cell transmission, which is a strategy for viruses to evade *in vivo* immunosurveillance, such as the extracellular neutralization effect. We currently do not know how the K394R mutation reduced the neutralizing potency of the prefusion F-induced antibodies in mouse serum. Several possible causes could be responsible for this: (i) the K394R variant has higher fusion activity during the viral entry, which narrows the time window for antibodies to neutralize the virions; (ii) induction of the K394R mutation may lead to the antigenic loss for neutralizing antibodies; and (iii) enhanced membrane fusion activity facilitates intracellular transmission for progeny virions between neighboring cells. These characteristics of the K394R variant highlight the importance of identifying alternative inhibitors against this variant. CL-A3-7 is a caffeoylquinic acid analog designed from the parent compound 3,4-DCQAME, a natural RSV fusion inhibitor identified in our previous investigations. Structural optimization improved the stability of this molecule and its potential binding mode with the trimeric prefusion RSV F. Consequently, the K394R variant elicited by 3,4-DCQAME did not show resistance to CL-A3-7. In addition, the escaped resistant virus did not emerge after culturing WT RSV for 16 passages in the presence of CL-A3-7, suggesting a low-drug resistance risk of CL-A3-7. Importantly, the combination treatment of CL-A3-7 with several RSV F inhibitor candidates (e.g., BMS-433771 and JNJ-53718678) dramatically reduced the risk of viral resistance induced by these inhibitors. These findings indicate that combination treatment using CL-A3-7 could be an alternative option to minimizing drug resistance risk, such as K394R-associated resistance, during anti-RSV therapy. However, we could not completely understand how the structural modifications conferred CL-A3-7 abilities against the K394R variant. Further structural biology studies are required to clarify this issue.

During the process of RSV entry into host cells, the virions initially adsorb to the cell membrane, followed by interaction with the corresponding F protein receptors to initiate virus–cell fusion. The cellular receptors and cofactors involved in RSV entry are not yet fully understood. Nucleolin has an important role during RSV entry. However, nucleolin is mainly distributed in the nucleus and widely expressed in almost all mammalian cells, with no obvious tissue specificity. The process by which it is transferred to the cell surface and mediates RSV-specific infection of human respiratory epithelial cells remains to be elucidated. Recent studies have demonstrated that IGF1R interacts with the RSV fusion protein to activate protein kinase C-ζ, which can recruit nucleolin to the host cell surface (28). RSV infects human respiratory epithelial cells through the synergistic effects of IGF1R and nucleolin. Therefore, interfering with the binding of the fusion protein to IGF1R can block the transfer of nucleolin protein from the nucleus to the cell membrane, thereby inhibiting RSV entry into host cells. Our previous study demonstrated that 3,4-DCQAME, the parent compound of CL-A3-7, blocked the binding of RSV F to the cell membrane. However, it did not disrupt the interaction between the F protein and the nucleolin receptor (27). This study demonstrated that the interactions between IGF1R and RSV F or F-K394R were inhibited by CL-A3-7. In addition, CL-A3-7 reduced viral loads and alleviated pathological changes and inflammatory responses in the lungs and tracheal tissues of RSV-infected mice. Current fusion inhibitors primarily hinder the conformational changes of the viral F protein to prevent RSV entry. This study provided promising evidence that blocking the interaction between the F protein and its cellular receptor could be an alternative strategy for anti-RSV drug development.

## MATERIALS AND METHODS

### Cells, viruses, and antiviral compounds

The human epithelial cell line HEp-2, human lung epithelial cell line A549, human normal bronchial epithelial cell lines BEAS-2B and 16HBE, human embryonic kidney cell line HEK293T, and *Aedes albopictus* mosquito cells line C636 were obtained from the American Type Culture Collection (ATCC, USA). HEp-2, BEAS-2B, 16HBE, and HEK293T cells were grown in Dulbecco’s modified Eagle’s medium (DMEM) containing 10% fetal bovine serum (FBS). C636 cells were cultured in minimum essential medium (MEM) supplemented with 10% FBS. The RSV A2 strain (ATCC, VR-1540) and long strain (ATCC, VR-26) were obtained from Wuhan University, China. RSV A2 variant harboring a K394R mutation in the F glycoprotein was isolated and identified in our previous study (27). All viruses were propagated in HEp-2 cells and viral titers were measured using TCID_50_ and plaque assays (34, 35). Ribavirin, heparin, and BMS-433771 were purchased from Sigma-Aldrich, Inc. TMC-353121, AK-0529, RV-521, JNJ-53718678, and zelicapavir were purchased from MedChem Express, Inc. All the inhibitors were dissolved according to the manufacturer’s instructions and stored at −20°C for subsequent experiments.

### Preparation of CL-A3-7

Compound CL-A3-7 was designed and synthesized by our group. Applied cyclohex-2-en-1-one as starting material, eight steps reactions including reduction, benzyl protection, di-hydroxyl oxidation, di-methylsulfation, di-azide substitution, Staudinger reaction, di-acylation, and benzyl de-protection were carried out. A mixture containing CL-A3-7 was obtained thereafter. After purified by fast liquid chromatography with dichloromethane/methanol (*V*_DCM_:*V*_CH3OH_ = 25:1, *R_f_* = 0.61), combined with RP-HPLC, the pure CL-A3-7 was collected in red solid. Purity >98%. $[\alpha {]}_{D}^{20}$ = −216° (*c* = 0.5, MeOH). ^1^H NMR (400 MHz, CDCl_3_) δ 7.85 (dt, *J* = 15.6, 3.5 Hz, 2H), 7.71 (s, 1H), 7.54–7.43 (m, 2H), 7.31–7.26 (m, 2H), 7.01–6.87 (m, 2H), 6.74 (d, *J* = 58.1 Hz, 1H), 6.48–6.29 (m, 2H), 4.44 (d, *J* = 2.2 Hz, 1H), 4.25 (s, 1H), 4.16 (d, *J* = 2.3 Hz, 1H), 1.91–1.68 (m, 6H); ^13^C NMR (100 MHz, CDCl_3_) δ 166.00 (s), 165.63 (s), 164.10 (s), 161.58 (d, *J* = 1.7 Hz), 138.92 (s), 138.35 (s), 131.27 (d, *J* = 3.8 Hz), 128.79 (d, *J* = 8.6 Hz), 125.50 (d, *J* = 9.5 Hz), 124.17 (s), 123.52 (s), 120.76 (s), 120.52 (s), 115.36 (s), 115.15 (s), 69.80 (s), 52.30 (s), 49.37 (s), 31.55 (d, *J* = 3.8 Hz), 29.30 (s), and 17.58 (s). HR-ESI-MS (*m/z*): calcd for C_24_H_23_N_2_O_3_F_2_Br_2_ [M + H]^+^ 583.0051, found: 583.0050.

### Plasmids and purification of recombinant proteins

DNA sequences encoding codon-optimized RSV F or RSV F with the K394R mutation were cloned into the pcDNA3.1(+) vector. pT7-Luc, pCAG-T7 Pol, and pRL-TK recombinant plasmids were constructed as described previously (18). The extracellular domain of the human IGF1R protein (Met 1−Asn 932) was expressed by Sino Biological Co., Ltd (Beijing, China) using HEK293 cells. DS-Cav1, F_(ecto)_-WT (1–513 aa), and F_(ecto)_-K394R (F_(ecto)_-WT with substitution of Lys394 with Arg394) were expressed and purified as described previously (36). Briefly, the DNA sequence encoding DS-Cav1, F_(ecto)_-WT, or F_(ecto)_-K394R fused with the T4 fibritin trimerization domain was cloned into the pcDNA3.1(+) vector with a C-terminal polyhistidine tag and transiently transfected into HEK293T cells. At 5 dpi, the cell supernatants were collected, centrifuged, passed over a Ni-NTA column, and eluted with a buffer containing 300 mM imidazole, 50 mM Tris-HCl, and 150 mM NaCl (pH 8.0). The fractions were concentrated, purified, and lyophilized in sterile phosphate-buffered saline (PBS; pH 7.4).

### Immunofluorescence staining

HEp-2, 16HBE, BEAS-2B, and HEK293T cells were seeded in 96-well plates 1 day before viral infection or transfection. For the viral infection assay, the cells were inoculated with RSV A2 or RSV A2 containing F-K394R mutation in the presence or absence of CL-A3-7. At 48 hpi, the cells were fixed with 4% paraformaldehyde in PBS for 20 min and permeabilized with 0.1% Triton X-100 in PBS for 10 min. After washing three times with PBS, the cells were blocked for 30 min with 4% bovine serum albumin (BSA) in PBS and then incubated with motavizumab (1: 500, Creative Biolabs, TAB-709) overnight at 4°C, followed by staining with Alexa Fluor 488-conjugated anti-human antibody (1:500, Thermo Fisher Scientific, A11013) for 2 h at room temperature (RT; 25°C). Nuclei were stained with 4, 6-diamidino-2-phenylindole (DAPI) for 10 min, and the cells were washed three times with PBS. Images were acquired using a confocal laser scanning microscope (Zeiss LSM800). In the transfection assay, plasmids expressing F, F-K394R, or IGF1R were transfected into HEK293T cells using a Lipofectamine 6000 kit (Beyotime, C0526).

### Plaque assay

HEp-2 cells were seeded in 24-well plates 1 day before viral infection. The cells were inoculated with the WT or the K394R variants for 2 h at 37°C. For plaque size comparison, the starting titers used for each virus were 50 PFU per well. The cell culture medium was removed, and the cells were washed two times with PBS. The cells were then overlaid with 500 µL of 1.5% agarose in a maintenance medium (2% FBS in DMEM). Once the agarose solidified, 500 µL of maintenance medium was added to each well, followed by incubation at 37°C for 5 days. The cells were fixed with 4% paraformaldehyde in PBS for 4 h and stained with 1% crystal violet for 30 min at RT. The plaques in each well were photographed under a microscope and analyzed using ImageJ software.

### Cell–cell fusion assay

A cell–cell fusion assay was conducted as described previously (18). Briefly, HEK293T cells were seeded into six-well plates 1 day prior to transfection. The cells were cotransfected with plasmids encoding RSV F or RSV F-K394R and pT7-Luc plasmids using a Lipofectamine 6000 kit (Beyotime, C0526). Another group of cells was cotransfected with pRL-TK and pCAG-T7 Pol plasmids. At 24 h post-transfection, the transfected cells were detached, mixed in equal proportions, and then reseeded into 96-well plates in the presence of dimethyl sulfoxide (DMSO), CL-A3-7, or ribavirin. At 48 h after transfection, firefly luciferase and renilla luciferase activities were detected using the Dual-Lumi Luciferase Reporter Gene Assay Kit (Beyotime, RG088S).

### RT-qPCR assay

Total RNAs from HEp-2 cells or lung homogenates of mice were extracted using an RNAprep Pure tissue kit (TIANGEN, DP431) or a FineProtect Universal RNA Kit (GENFINE, R203). The purified RNA (1 µg) was used as the template to synthesize cDNAs using a PrimeScript RT Reagent Kit with gDNA Eraser (Takara, RR047A). cDNAs were prepared for quantitative real-time PCR analysis using SYBR Green Master Mix (Takara, RR820A), according to the manufacturer’s protocols. Fluorescence signals were acquired using a Light Cycler 480 system (Roche, Basel, Switzerland). The following primer sequences were used: F forward, 5′-GCAAAGCACACCGGCAACCA-3′ and reverse, 5′-GCCACTGGCGATTGCAGATCCA-3′; NS1 forward, 5′-GGGCAGCAATTCGTTGAGTA-3′ and reverse, 5′-AGCACTGGCATTGTTGTGAA-3′; NS2 forward, 5′-TTGATGAAAGACAGGCCACA-3′ and reverse, 5′-TATCGGCATAGGGAAAGTGC-3′; P forward, 5′-TTTGCTAAGACTCCCCACCGTA-3′ and reverse, 5′-CTTACTACCCAAGGACATAGCCAAC-3′; GAPDH forward, 5′-GATTTGACCTTAGTACAAGGAGATAA-3′ and reverse, 5′-AGACAAGTAGACCAATGGAATAGAA-3′; IL-2 forward, 5′-GGTGAGCATCCTGGGGAGTTT-3′ and reverse, 5′-CTCTACAGCGGAAGCACAGCA-3′; IL-4 forward, 5′-GTTTGGCACATCCATCTCCG-3′ and reverse, 5′-TAGTTGTCATCCTGCTCTTCTTTC TC-3′; IL-6 forward, 5′-CACCAGCATCAGTCCCAAGAAG-3′ and reverse, 5′-TGGAGCCCACCAAGAACGA-3′; IFN-γ forward, 5′-GCCTGATTGTCTTTCAAGACTTCAA-3′ and reverse, 5′-TAACTCAAGTGGCATAGATGTGGAA-3′; TNF-α forward, 5′-CTCAGGGAAGAATCTGGAAAGGT-3′ and reverse, 5′-GCACCACCATCAAGGACTCAA-3’.

### Neutralization assay

HEp-2 cells were seeded into 96-well plates one day before viral infection. Serially diluted sera from immunized mice were preincubated with the WT or the K394R variant for 1 h, and 100 µL of the mixtures was added to cells. As a control, the cells were infected with the virus without serum. Viral stocks in each well were collected at 48 hpi and subjected to viral titer detection using TCID_50_ assay.

### Molecular docking analysis

Small-molecule compounds were prepared by Ligprep (Schrödinger) and converted into 3D structures using the OPLS4 force field. Then, Ligprep generated the expected ionized forms and tautomers at a target pH of 7.0 ± 2.0, and added or removed hydrogens to achieve charge neutrality. The 3D structures of RSV F (PDB, 7UJA) and F-K394R were modeled from the residue and loop mutations of Biologics in Maestro. Water molecules and hetero-atoms in the proteins were removed. Hydrogen atoms were added to the proteins, including correction and refinement of the structure using the OPLS4 force field. Induced Fit Docking (IFD) was performed to accurately predict the correct binding mode of the inhibitors within the active pocket of the proteins. This methodology included glide docking, prime refinement, and glide redocking to generate the best-docked poses with △*E* < 30 kcal/mol. The docking box was settled using active-site residues and the results yielded an IFD score for each output pose. Finally, the data analysis was performed with PyMol 2.6.0.

### Viral growth kinetics

HEp-2, A549, 16HBE, and BEAS-2B cells were grown in 96-well plates and inoculated with RSV in the presence of CL-A3-7, BMS-433771, TMC-353121, JNJ-53718678, AK-0529, zelicapavir, or DMSO, respectively. The cells and supernatants were harvested at various time points. The collected cells were freeze–thawed two times to release virions and then centrifuged to remove cellular debris. The viral titer and RNA load were determined using the TCID_50_ assay and RT-qPCR, respectively.

### Time-of-addition assay

HEp-2, 16HBE, and BEAS-2B cells were seeded into 96-well plates and incubated overnight. Cell culture medium containing CL-A3-7 was added at specified time points (−2, 0, 2, 4, 6, 10, 14, 22, 30, and 38 h) before (−2 and 0 h) or after (2, 4, 6, 10, 14, 22, 30, and 38 h) the viral infection. At 48 hpi, the viral suspensions were collected and subjected to viral titer detection.

### Attachment assay

HEp-2, BEAS-2B, and 16HBE cells were dissociated and centrifuged for 5 min at 4°C. The pelleted cells were resuspended in a 4°C precooled virus suspension containing CL-A3-7 or heparin. The cells were then shaken for 1 h at 4°C, followed by centrifugation at 1,500 rpm at 4°C for 3 min, and washed two times with PBS to remove the unbound virions. The cells were fixed with 4% paraformaldehyde in PBS for 15 min at 4°C. After washing two times with PBS, the cells were incubated with motavizumab for 2 h at RT and then stained with an Alexa Fluor 488-conjugated secondary antibody (Thermo Fisher Scientific, A11013) for 1 h. Finally, the cells were washed three times with PBS and detected by flow cytometry. Data were analyzed using FlowJo v.10.0.

### R18-RSV fusion assay

Purified virions were resuspended in DMEM medium and stained with octadecyl rhodamine B chloride (R18; Thermo Fisher Scientific) at a final concentration of 10 µM for 30 min at RT, and then passed through a 0.22-µm filter syringe filter (Millipore). HEp-2 cells were preincubated with CL-A3-7 (40 µM), ribavirin (40 µM), or heparin (4  µM) before R18-RSV infection. At 2 hpi, the cells were fixed with 4% paraformaldehyde for 20 min and washed three times with PBS. The fluorescence intensity of R18 was detected by flow cytometry (BD Biosciences). Data were analyzed using FlowJo version 10.

### Enzyme-linked immunosorbent assay

To determine the binding of IGF1R to RSV F, Maxisorp (Nunc) ELISA plates were coated with IGF1R protein (SinoBiological, 10164-H49H1-B) for 12 h at 4°C. The plates were blocked with a 4% (wt/vol) solution of BSA in PBS for 2 h at RT. Serial dilutions of F(ecto)-WT or F(ecto)-K394R with CL-A3-7 (20 µM) or DMSO were added to the plates and incubated for 2 h at RT. Specific antibodies against the RSV F protein (Abcam, ab94968) and horseradish peroxidase (HRP)-conjugated secondary antibody (Abcam, ab6759) were used to stain F-WT and F-K394R in each well. After washing three times with PBS, tetramethylbenzidine (TMB) substrate was added to measure the peroxidase activity, and the reaction was stopped when the color developed to the desired depth. Absorbance at 450 nm was recorded using a microplate reader.

### Colocalization assay

HEK293T cells were seeded in confocal dishes 1 day before transfection. Plasmids encoding RSV F or RSV F with K394R mutation were transfected into the cells using Lipofectamine 6000. At 48 h post-transfection, soluble IGF1R (20 µg/mL) was premixed with CL-A3-7 (20 µM) for 1 h. Then, 100 µL of the mixtures was added to transfected cells for 5 h and fixed with 4% paraformaldehyde in PBS for 15 min, followed by washing three times with PBS and permeabilized with 0.1% Triton X-100 for 10 min. The cells were blocked for 30 min with 4% BSA in PBS and then incubated with humanized motavizumab (Creative Biolabs, TAB-709) and rabbit anti-IGF1R primary antibodies (Bioss, bs-4985r) overnight at 4°C, followed by staining with Alexa Fluor 488 and 594 nm secondary antibodies for 2 h at RT. The nuclei were stained with DAPI for 10 min, and the cells were washed two times with PBS. Images were acquired using a confocal laser scanning microscope (Zeiss LSM800). In addition, colocalization of sIGF1R with F-WT or F-K394R was quantitatively analyzed by flow cytometry. The experimental procedure was performed as described above using cells without DAPI staining.

### Immunoprecipitation and western blot assay

HEK293T cells were transfected with pcDNA3.1 plasmids expressing IGF1R, F-WT, or F-K394R. At 6 h after transfection, the Opti-MEM medium was replaced with a fresh medium containing CL-A3-7 (20 µM). The transfected cells were lysed at 30 h and the supernatants of cell lysates were collected after centrifuge at 4°C, 12,000 rpm for 15 min. Protein A/G beads were preincubated with anti-RSV F antibody (Abcam, ab94968) for 12 h at 4°C. The protein lysates were incubated with the antibody–bead complex for an additional 24 h. After washing three times with PBS, the beads were mixed with an equal volume of 2× sodium dodecyl sulfate-polyacrylamide gel electrophoresis (SDS-PAGE) loading buffer and boiled for 10 min at 95°C. Protein supernatants were separated by SDS-PAGE and transferred onto polyvinylidene fluoride (PVDF) membranes. The blotted proteins on the PVDF membranes were incubated with anti-IGF1R (HUABIO, R1310-6), anti-RSV F (Abcam, ab94968), and anti-GAPDH (Cell Signaling Technology, D4C6R) antibodies, and then detected with chemiluminescent agents using a chemiluminescence imaging system (Amersham Imager 600, GE Healthcare).

### Microscale thermophoresis assay

Purified RSV F-WT and F-K394R proteins were labeled with RED-NHS dye (NanoTemper Technologies, MO-L011) for 30 min at RT. Labeled proteins (50 nM, final concentration) were pretreated with CL-A3-7 (40 µM) or DMSO and then mixed with IGF1R protein at serial dilutions. The reaction buffer consisted of PBS and various concentrations of IGF1R ranging from 30.6 pM to 2 µM. Microscale thermophoresis was performed using the Monolith NT.115 instrument (NanoTemper Technologies). *K*_*D*_ values were calculated using MO. Affinity Analysis software.

### Animal experiments

BALB/c mice were purchased from SPF Biotechnology Co., Ltd. (Beijing, China). To assess *in vivo* viral pathogenicity, BALB/c mice (female, 4 weeks old) were intranasally inoculated with wild-type RSV A2 (1 × 10^6^ plaque-forming units [PFU]) or the K394R variant (1 × 10^6^ PFU) under isoflurane anesthesia. At 1, 3, 5, and 7 dpi, five or six mice from each group were euthanized. Lung and tracheal tissues were prepared for H&E staining, histological scoring, and viral RNA quantification. For the preparation of serum from prefusion F-immunized mice, BALB/c mice (female, 6 weeks old) were intramuscularly immunized with 5 µg of DS-CaV1 or vehicle on days 0, 14, and 28. Before vaccination, soluble DS-CaV1 proteins were prepared in PBS at a 1:1 ratio with AddaVax (InvivoGen). Sera from blood of the mice were collected on days 24 and 38 after the first immunization. The prepared sera were diluted to assess its ability to neutralize RSV infection in HEp-2 cells.

To evaluate the *in vivo* anti-RSV activity of CL-A3-7, BALB/c mice (female, 4 weeks old) were infected with the K394R variant (5 × 10^5^ PFU) intranasally under isoflurane anesthesia. The mice were treated with CL-A3-7 (20 and 40 mg/kg) or vehicle (10% DMSO, 90% PEG-400) intranasally once daily. At 5 dpi, the mice were euthanized, and the lung tissues of infected mice were collected for detection of viral RNA loads and titers. H&E staining and immunostaining of the tissue sections were also performed.

### Micro-CT imaging

The respiratory tracts and lungs of the mice were imaged at 5 dpi using a Super Nova CT scanner (snc-100). Under anesthesia induced and maintained with isoflurane, the mice were placed in the imaging chamber and scanned at 60 kV, 500 μA with a field of view of 90 mm for 4 min. After scanning, the lung images were reconstructed and analyzed using the Avatar system software. Qualitative and semi-quantitative analyses of the lung images were performed on three mice for each group. The CT severity score was measured to determine the severity of the lung abnormalities.

### Lung lesion scoring of H&E staining

The severity of bronchitis or bronchiolitis, hemorrhage, alveolar damage, and the area of large type II pneumocytes were arbitrarily scored using a four-tiered system: 0 (negative), 0–1 (weak), 1–2 (moderate), 2–3 (moderately severe), and 3–4 (severe). In particular, “large type II pneumocytes” are one of the histopathological features of RSV infection. For assessment of the area of large type II pneumocytes, the presence of more than five large type II pneumocytes with a nuclear diameter of 10 µm per 0.04 mm^2^ was delineated and measured using CaseViewer 2.4 software. The total red-colored indicated areas were counted as the percentage of the total lung lobe area.

### Statistical analysis

Data are presented as mean ± standard deviation (SD). Statistical analyses were performed using GraphPad Prism software version 8.0 (GraphPad Software Inc., La Jolla, CA, USA). The statistical differences between the compared groups were calculated using a two-tailed Student’s *t* test unless otherwise stated. *P* values < 0.05 indicate statistically significant differences between the compared groups.

## Data Availability

All data needed to evaluate the conclusions in the paper are present in the paper and/or the supplementary material.

## SUPPLEMENTAL MATERIAL

Supplemental Material mbio.01385-24-s0001.docx Fig. S1 to S7.

ASM does not own the copyrights to Supplemental Material that may be linked to, or accessed through, an article. The authors have granted ASM a non-exclusive, world-wide license to publish the Supplemental Material files. Please contact the corresponding author directly for reuse.
