# Supplementary material for: A new mechanism of respiratory syncytial virus entry inhibition by small-molecule to overcome K394R-associated resistance
Source: mBio. 2024 Aug 20;15(9):e01385-24. doi: 10.1128/mbio.01385-24 (PMC11389407; doi:10.1128/mbio.01385-24)
Supplement: Supplemental Material — Fig. S1 to S7. [file mbio.01385-24-s0001.docx]

Supplementary Materials for

**A new mechanism of respiratory syncytial virus entry inhibition by small-molecule to overcome K394R-associated resistance**

Qiaoyun Song *et al.*

*Corresponding author. Email: tangw@jnu.edu.cn (Wei Tang), wangying_cpu@163.com (Ying Wang), thrchen@jnu.edu.cn (Heru Chen), chywc@aliyun.com (Wencai Ye).

**This PDF file includes:**

Figures. Supplementary Fig.1 to Fig.7


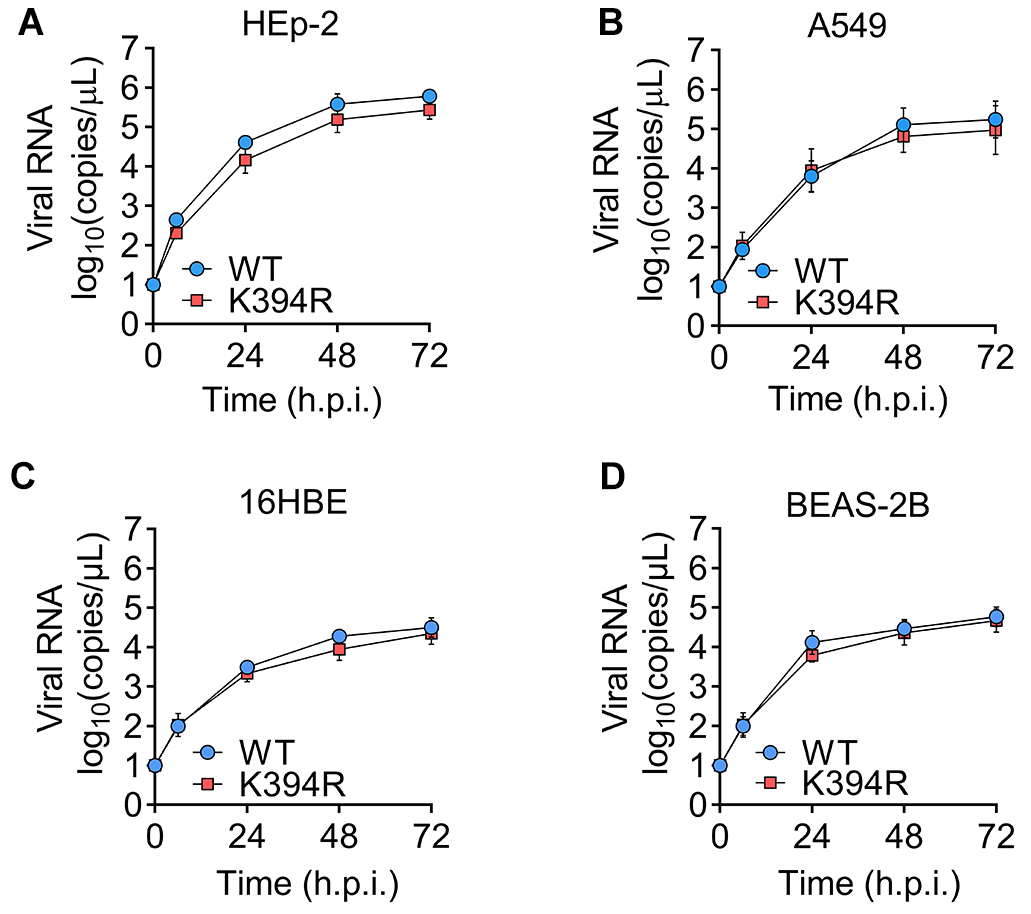


**Supplementary Fig. 1. Viral growth kinetics of WT and K394R variant in different types of cells.** Viral RNA loads in HEp-2 (**A**), A549 (**B**), 16HBE (**C**), and BEAS-2B (**D**) cells were detected by RT-qPCR assay. Data are mean ± SD, *n* = 3 biological replicates.

**
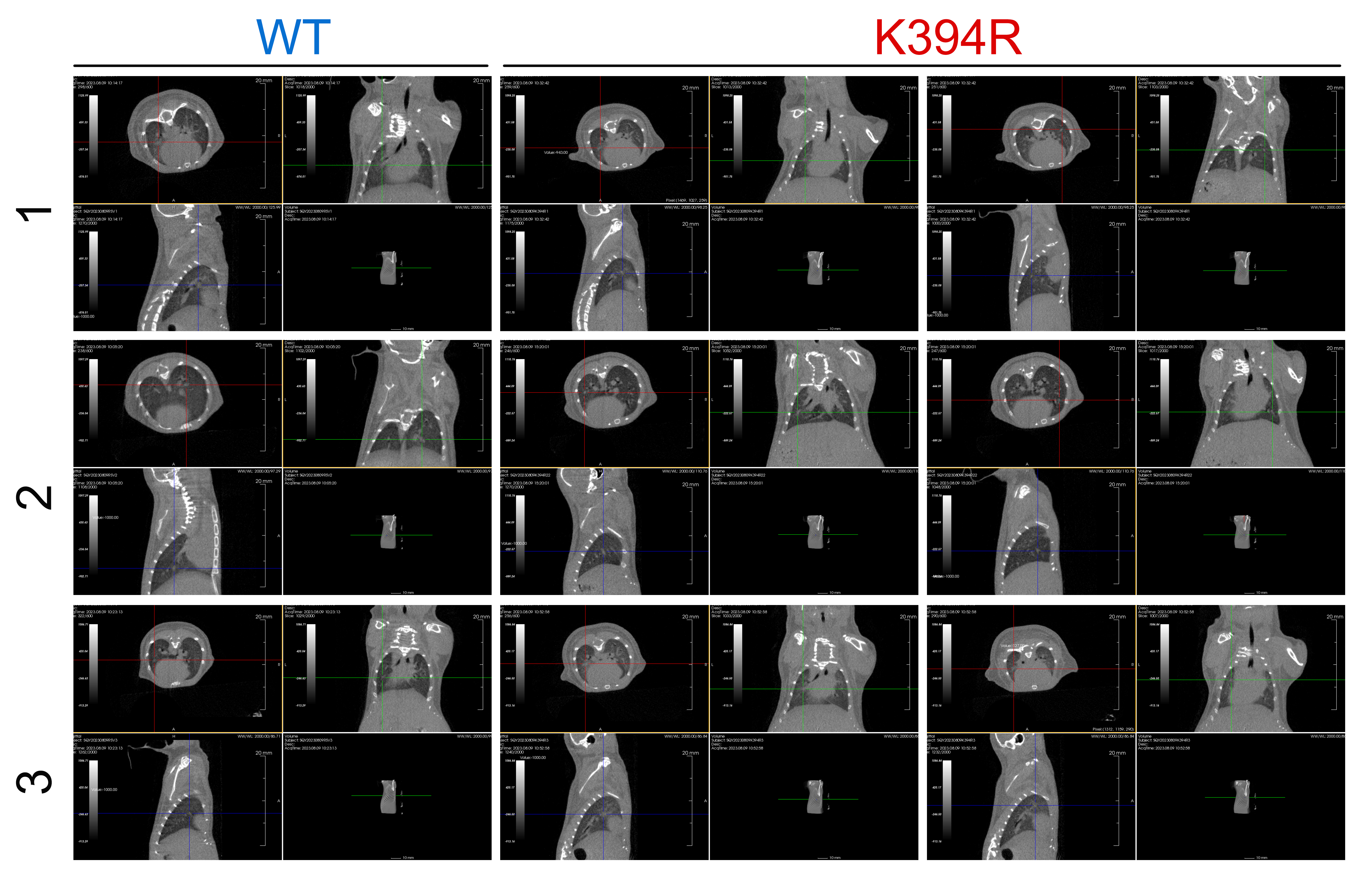
**

**Supplementary Fig.** **2.** **Micro-CT scans of the lung organs of infected mice.** Direct axial and coronal images of the lungs of WT-infected mice (n = 3) and K394R-infected mice (n = 3) are shown. Lung abnormalities are indicated at crossed sites of color lines.

**
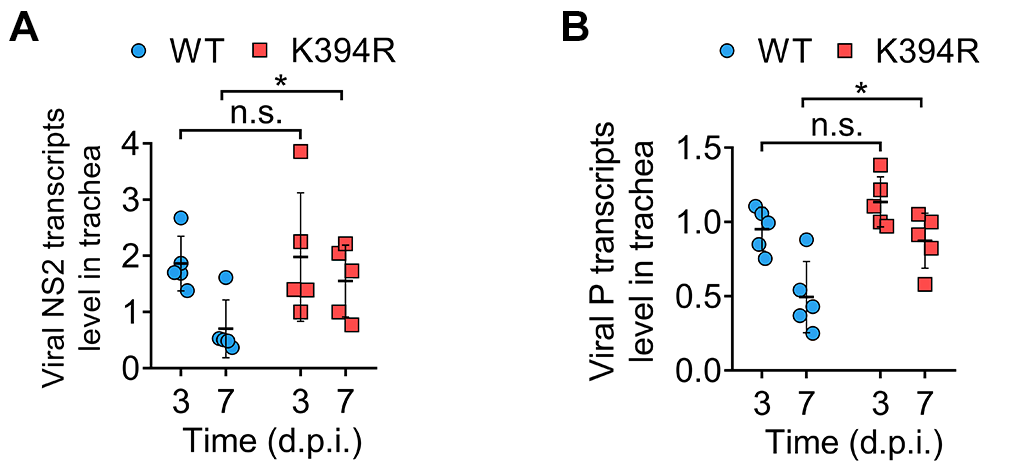
**

**Supplementary Fig. 3. Viral RNA loads in the tracheas of infected mice.** Trachea homogenates of WT- and K394R variant-infected mice were prepared at 3 and 7 dpi. (**A** and **B**) mRNA levels of viral non-structural protein 2 (NS2) and phosphoprotein (P) in the tissue homogenates of mice were detected using RT-PCR. Data are mean ± SD (*n* = 5). The two-tailed Student’s t test was used to measure the statistical difference between groups. *, *P* < 0.05.


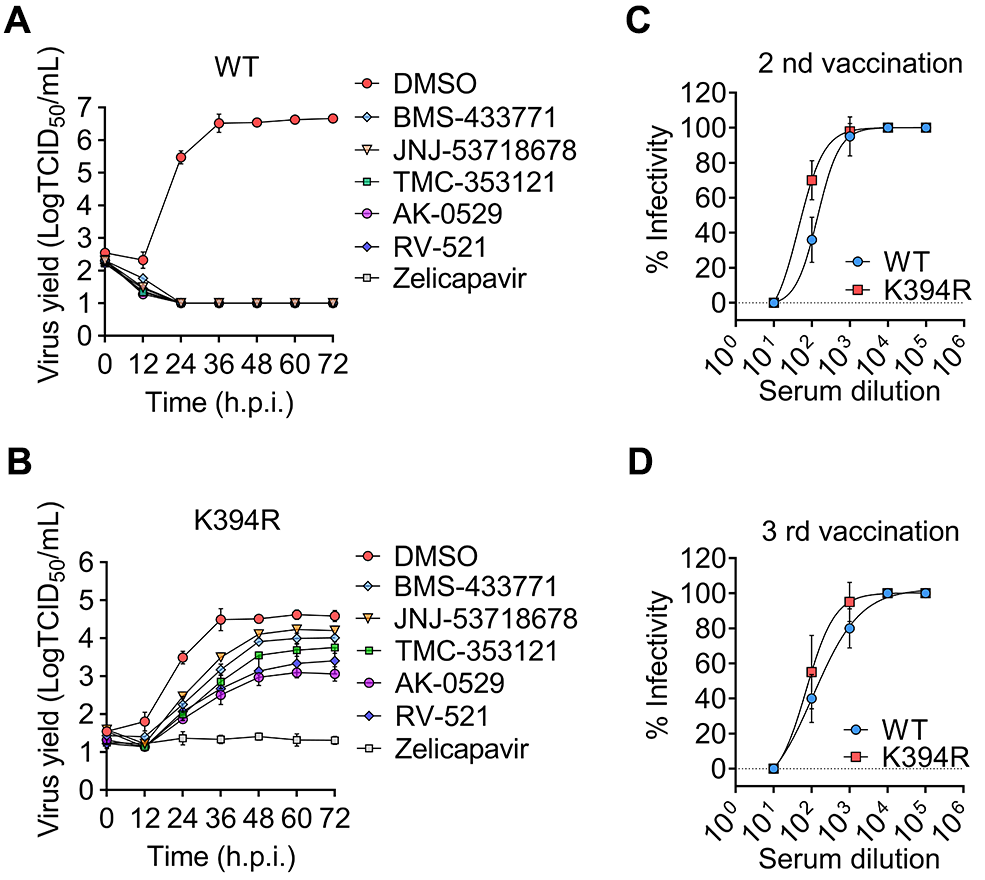


**Supplementary Fig.** **4. Inhibition of WT and K394R variant infections by RSV inhibitors and mouse serum after prefusion F immunization.** Growth kinetics of the WT (**A**) and the K394R variant (**B**) in HEp-2 cells that were treated with DMSO, RSV fusion inhibitors (BMS-433771, JNJ-53718678, TMC-353121, AK-0529, and RV-521), or RSV nucleoprotein (N) inhibitor. All the inhibitors were tested at 20-fold IC_50_. Data are mean ± SD, *n* = 3 biological replicates. (**C** and **D**) Neutralization assay using the mouse sera vaccinated by DS-Cav1. HEp-2 cells were inoculated with the WT or the K394R variant in the presence of diluted sera from 2 ^nd^ or 3 ^rd^ vaccinated mice. Data are mean ± SD (*n* = 5).


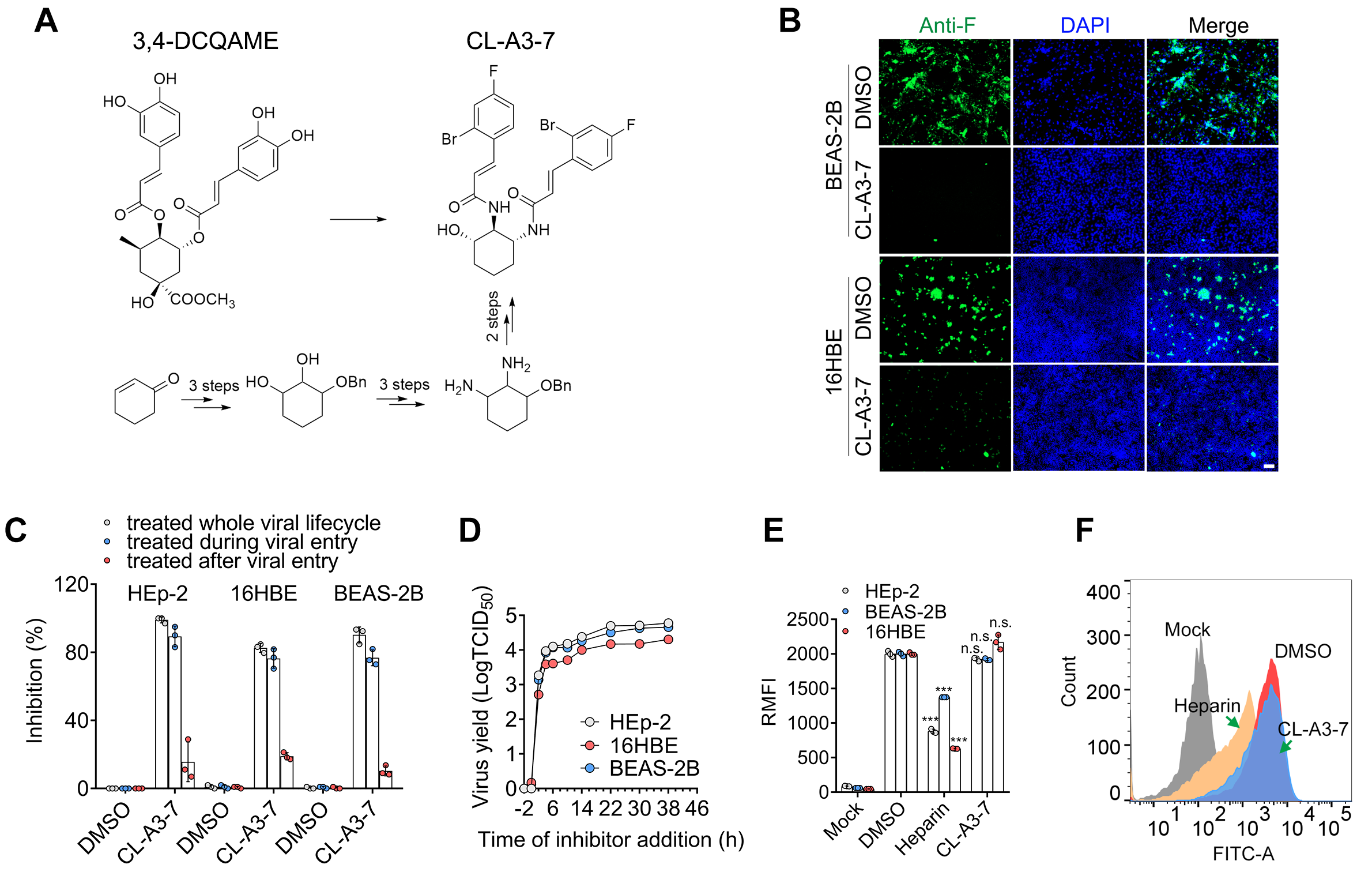


**Supplementary Fig.** **5. Inhibitory effect of CL-A3-7 on RSV entry.** (**A**) Preparation of CL-A3-7. (**B**) The expression levels of RSV F protein in BEAS-2B and 16HBE cells that were treated with CL-A3-7 (20 μM) or DMSO. Bar, 100 μm. (**C**) Inhibition of RSV entry by treatment of CL-A3-7 (20 μM) during (0−2 h), after (2−40 h) viral entry, or whole viral lifecycle (0−40 h). Data are mean ± SD, *n* = 3 biological replicates. (**D**) Virus yields in RSV-infected cells that were treated with CL-A3-7 (20 μM) at indicated hours after infection using TCID_50_ assay. (**E**) The numbers of virions binding on the cell surface in the presence of heparin (4 μM), CL-A3-7 (40 μM), or DMSO using flow cytometry. RMFI, relative mean fluorescence intensity. Data are mean ± SD, *n* = 3 biological replicates. Statistically significant differences in comparison between CL-A3-7- or heparin- and DMSO-treated cells are indicated. The two-tailed Student’s t test was used to measure the statistical difference between groups. ***, *P* < 0.001. (**F**) Representative histograms showing the attachment of virions on the BEAS-2B cell surface.


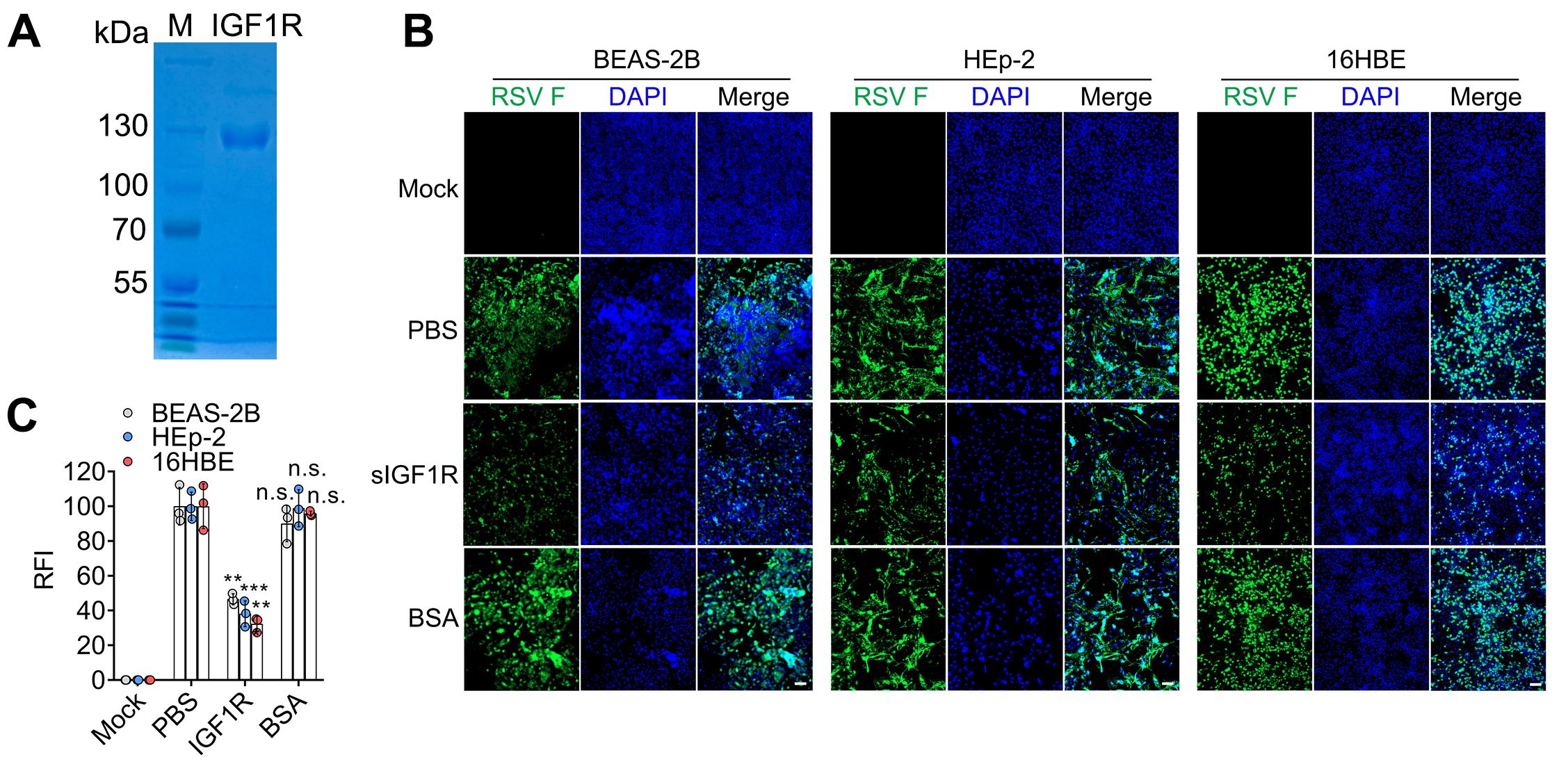


**Supplementary Fig. 6. Neutralizing effect of IGF1R on RSV infection. (A)** SDS-PAGE analysis of the purified IGF1R. (**B**) Inhibitory effect of the purified IGF1R on RSV infection. RSV (WT) were pre-mixed with IGF1R (0.2 μM) or BSA (0.2 μM) for 20 min at RT, and then the mixtures were added to BEAS-2B, HEp-2, and 16HBE cells, respectively. At 40 h.p.i., RSV F proteins in the cells were stained with motavizumab and Alexa flour 488-conjugated secondary antibodies, followed by detection with a fluorescence microscope. Representative images are shown. Bar, 100 μm. **(C)** Relative fluorescence intensity (RFI) from each group of cells was analyzed (n = 3 to 4 per group). Statistically significant differences in comparison between PBS- and IGF1R- or BSA-treated cells are indicated. The two-tailed Student’s t test was used to measure the statistical difference between groups. **, *P* < 0.01.***, *P* < 0.001.


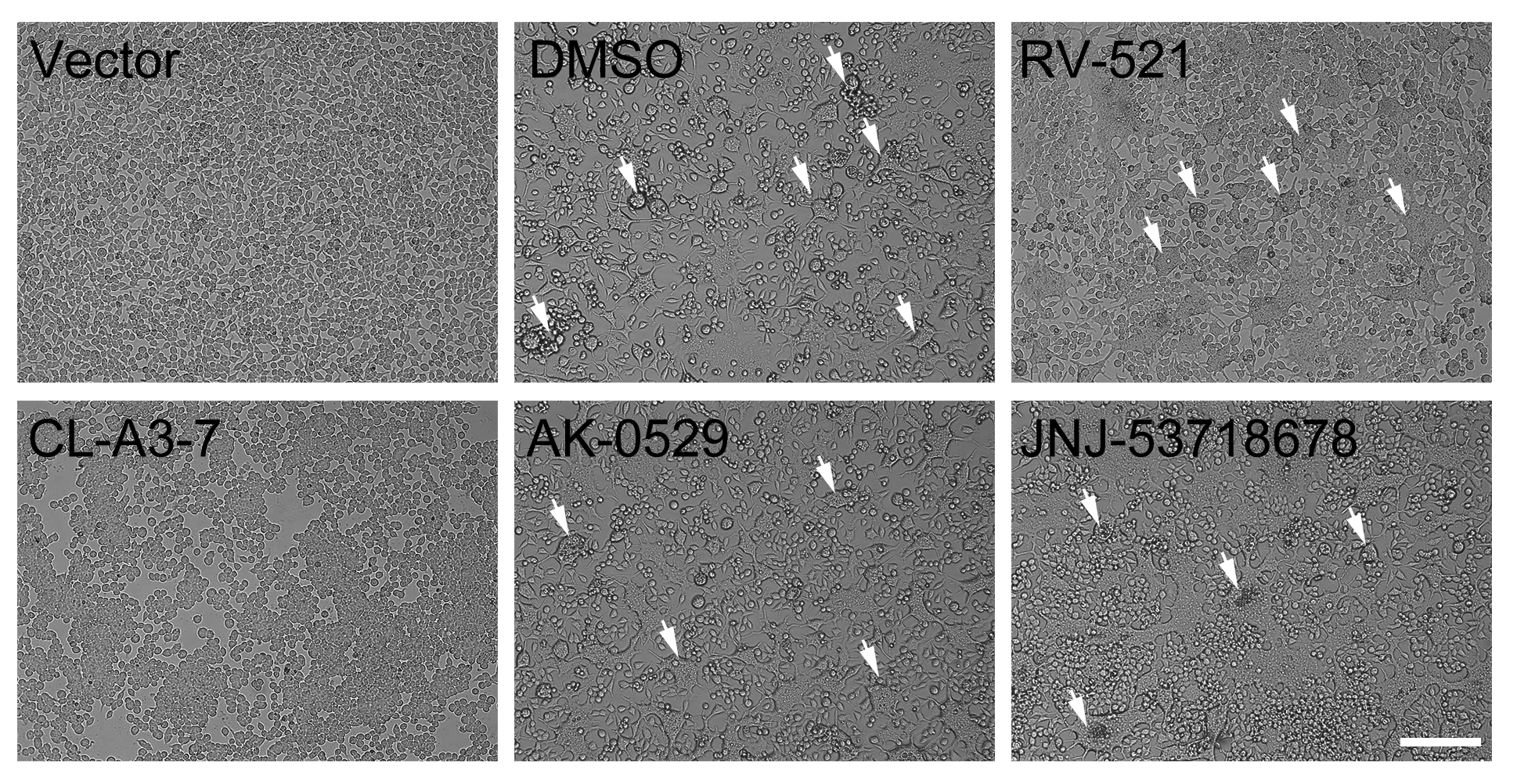


**Supplementary Fig.7. Resistance of F-D489Y to RSV fusion inhibitors.** HEK293T cells were transfected with pcDNA3.1 or pcDNA3.1 encoding RSV F with D489Y mutation. At 6 h after transfection, the cells were treated with DMSO, CL-A3-7 (20 μM), or fusion inhibitors that were insensitive to RSV F-D489Y mutant as previously described. The concentrations of RV-521, AK-0529, and JNJ-53718678 used in the experiments are 28 nM, 84 nM, and 17 nM, respectively, which are approximately 20-fold higher than their IC_50_ values in inhibition of WT RSV infection. After incubation for 24 h, the cells were photographed under a microscope. The representative syncytia in the images are indicated as white arrows. Bar, 2 mm.
